# Environmental, social and governance performance and green transformation strategies for enterprises: Improving technical efficiency or expanding technological boundaries

**DOI:** 10.1371/journal.pone.0299767

**Published:** 2024-03-14

**Authors:** Dengyun Niu, Zhihua Wang

**Affiliations:** Business School, Shanxi Datong University, 037009, Datong, China; University of Calabria Department of Mechanical Engineering Energy Engineering and Management Engineering: Universita della Calabria Dipartimento di Ingegneria Meccanica Energetica e Gestionale, ITALY

## Abstract

Under the ongoing implementation of the “dual carbon” goal, research has focused on the impact of Environmental, Social, and Governance (ESG) initiatives on green innovation. However, few studies have analyzed in depth the mechanisms of ESG impact on green total factor productivity (green TFP). Here, we explored the impact of ESG performance on green TFP, green technical efficiency, and green technological progress using A-share listed companies in China’s Shanghai and Shenzhen stock markets from 2011 to 2021. The results show that good ESG performance can significantly improve the green TFP of enterprises, and that this effect is more prominent in industries with lower environmental risks and enterprises in the growth and maturity stages. We identified the importance of the psychological account path and propose that the promotional effect of ESG performance on green TFP mainly derives from improving green technical efficiency, rather than from expanding green technological boundaries. These findings have practical implications for guiding companies to implement ESG concepts, strengthening the synergistic role of government regulation and professional supervision, and promoting micro-level implementation of innovation-driven and sustainable development strategies, thereby promoting high-quality development.

## 1. Introduction

Against the global backdrop of frequent extreme weather events and resource depletion, environmental issues have become an important constraint on economic development; consequently, the concept of sustainable development is reshaping the behavior of enterprises [1]. Environmental, Social, and Governance (ESG) represent the three dimensions used to measure the level of green and sustainable development of enterprises. China’s requirements for ESG information disclosure by listed companies are gradually becoming aligned with international standards. In 2018, the China Securities Regulatory Commission revised the “Guidelines for the Corporate Governance of Listed Companies,” clarifying the basic framework for ESG information disclosure. In 2021, this was followed by the revised “Guidelines No. 2 for the Content and Format of Information Disclosure of Companies Issuing Securities to the Public” that added new requirements for ESG information disclosure. In Hong Kong, the 2012 “Environmental, Social and Governance Reporting Guidelines” issued by the Hong Kong Stock Exchange recommended that listed companies disclose ESG information voluntarily; in 2016, some recommendations were elevated to a semi-mandatory disclosure level; and the 2019 version of the guidelines further expanded the scope of mandatory disclosure and implemented the principle of “disclose or explain.”

According to the 21st Century Capital Research Institute, as of August 2023 there were 1,771 A-share listed companies in China (33.78% of the total) that had independently disclosed ESG or social responsibility reports. This indicates that under the dual promotion of policies and the market, the willingness and level of ESG disclosure by listed companies in China have increased significantly. In response to the improvements made to the ESG system, the academic community has begun to discuss the connotations and measurements, influencing factors, economic consequences, and other aspects of ESG disclosure, evaluation, and investment, mainly focusing on the economic consequences of ESG. Numerous studies have found that ESG can help alleviate the financing constraints of companies [2], and good ESG performance can reduce capital costs [3,4] and enhance a firm’s ability to resist risks [5], thereby enhancing firm value [6,7].

Although existing studies provide a valuable foundation, research gaps remain that require further exploration. First, previous studies have discussed the positive effects of ESG on reducing production costs, increasing product value, and promoting green innovation [8,9], as well as the impact of company or industry factors on green total factor productivity (TFP) [10], but there is still a lack of research linking the two together. In the context of dual carbon goals and sustainable development, this study links ESG and green TFP to explore how green governance can empower high-quality development, and whether the market regulation generated by ESG can strengthen the sustainable development momentum of enterprises.

Second, existing studies mainly use industry or regional data to study the influencing factors or differential performance of green TFP [11], with relatively few studies at the enterprise level. This study used the SBM (Slacked-Based Measure) directional distance function to construct an indicator system for green TFP at the enterprise level, providing empirical evidence of the relationship between ESG and green TFP at the micro level.

Third, some scholars have focused on green TFP, but there is a lack of exploration of potential mechanisms and the exploration of pathways is not in-depth. After clarifying that ESG has a significant improvement effect on green TFP, this study further decomposed green TFP into green technical efficiency (GEC) and green technological progress (GTC) based on the Malmquist–Luenberge (ML) productivity index. As such, we were able to further clarifying whether the contribution of ESG to green TFP stems from the improvement of technological efficiency or the expansion of technical boundaries.

To bridge these research gaps, this study applied the perspectives of stakeholder theory and psychological account theory to Chinese A-share listed companies over the period from 2011 to 2021, empirically tested the effect of ESG performance on green TFP, and examined managers’ psychological accounts. Because enterprises in different stages of their life cycle and facing different environmental risks encounter significant differences in investment decisions and external environmental requirements, this study explored the heterogeneous effects of industrial environmental risks and enterprise life cycles, attempted to control for endogeneity issues, and conducted multiple robustness tests. Our findings provide a reference for identifying differences in green transformation strategies of enterprises, open the “black box” of ESG impacts on enterprise behavior, reveal the interaction mechanism between macro conditions and micro paths, and provide a measure of contextually matched theoretical support for ESG practices.

## 2. Data and methods

### 2.1 Hypotheses

China’s ESG construction started relatively late, and unlike Western countries that mainly rely on “top-down” policies to compel companies to disclose ESG information [12], Chinese corporate performance has been mainly evaluated by third-party institutions. According to stakeholder theory, enterprises are the sum of multilateral relationships. The core resources provided by stakeholders, including the credit resources provided by investors and creditors, the market competitiveness resources provided by suppliers and customers, and the political resources provided by government departments, are all essential for sustainable development. Whether enterprises can establish good relationships with stakeholders and satisfy their needs affects their development capabilities. A better ESG performance corresponds to a greater ability of a company to meet the needs of various stakeholders and public expectations [13,14]. This conveys a positive signal for enterprises to safeguard their stakeholders, which can not only alleviate financial constraints [15], but also enhance the confidence of stakeholders, improve their ability to obtain scarce resources [16], and ultimately empower high-quality development of enterprises and promote the improvement of green TFP.

Under the “dual carbon” target, stakeholders have gradually begun to pay attention to balancing economic development and the ecological environment. A higher environmental (E) performance in ESG ratings indicates that the company pays greater attention to environmental protection, can actively respond to government environmental regulatory requirements [17], uses renewable energy and clean equipment [18], shows improved pollution control and resource utilization efficiency, and has developed green environmental protection products [19]. This will convey a positive signal to the outside that the company attaches importance to sustainable development, which can meet the environmental demands of consumers and investors, as well as obtain more social public support, government subsidies, tax incentives, and financial resource support [20], thereby reducing the costs of green transformation and creating conditions for the improvement of green TFP.

In terms of social (S) aspects, enterprises are mainly evaluated for whether they have actively fulfilled their social responsibilities during the development process and whether they can effectively satisfy the interests of stakeholders. Companies with better social (S) performance based on ESG ratings usually support local economic development [21], donate to charitable organizations [22], attend to the protection of employee rights and interests, and have higher workplace safety, which can gain the trust and support of stakeholders [23], promoting effective communication and positive interaction between enterprises and stakeholders. At the same time, it also objectively aligns the interests of employees with those of enterprises, improves the satisfaction and work efficiency of employees, and promotes the improvement of green TFP.

Finally, in the context of separation of ownership and control, and conflicts between shareholders and management, large shareholders and small shareholders will both affect the sustainable development of enterprises. A better governance (G) performance implies greater completeness of internal governance mechanisms (e.g., supervision, balance, and feedback) and greater effectivity in balancing the relationship between stakeholders [24]. This can enhance the scientific decision-making of managers [25], make them attach importance to green transformation, reduce opportunistic tendencies and self-interested behaviors, and maximize the alignment of managers’ own interests, the interests of various stakeholders, and the overall interests of the enterprises [26]. This will help enterprises form long-term investment preferences and promote the improvement of green TFP. Based on these assumptions, the first hypothesis posited in this study.

H_1_: Good ESG performance can promote the improvement of green TFP of enterprises.

Psychological account theory in behavioral finance suggests that managers have certain psychological accounts when making investment decisions, which will inevitably lead to short-sightedness when choosing green transformation strategies even if a longer-term vision is involved. To examine short-sightedness caused by psychological accounts, this study decomposed green TFP into two aspects: green technical efficiency and green technological progress [27]. The former refers to approaching technological boundaries by optimizing factor allocation and improving management levels [28], and improving green TFP through efficiency improvement. The latter refers to expanding technological boundaries by promoting technological innovation, maintaining competitive advantages [29], and improving green TFP. According to psychological account theory, the stimulation and impact exerted on managers by a company’s ESG performance are affected by the psychological account, which may cause manages to pay greater attention to improving green technical efficiency improvements that require less investment, lower risk, and better short-term benefits, while avoiding technological progress strategies that require more investment, higher risk, and better long-term sustainability.

Specifically, managers tend to establish psychological accounts to compare the advantages and disadvantages of various decision-making options [30]. Moreover, the impact of loss is greater than that of an equal amount of gain [31], which causes managers to overweigh recent gains and losses in their strategic choices and renders them unable to apply a global perspective, resulting in non-optimal decisions [32] and short-term loss aversion. Although good ESG performance can improve a company’s green TFP, the short-sightedness caused by psychological accounts leads to predominantly improvement-oriented enhancements, such as optimizing the organizational structure and production processes [33], improving production and pollution control equipment, and enhancing resource allocation efficiency and management efficiency to meet regulatory environmental requirements. This is effective in the short term and can quickly promote the improvement of green TFP. However, from the perspective of long-term sustainable development, another outcome is maximized loss avoidance in decision-making [34], resulting in a lack of endogenous driving force to promote green technological progress and expand the boundaries of green technology [35]. Consequently, such managers may reduce investment in green innovation, thereby inhibiting improvements in green TFP. In summary, managers make long-term decisions based on the evaluation of short-term returns, which results in ESG performance mainly promoting the improvement of a company’s green technical efficiency rather than green technological progress. Based on this background, the second hypothesis posited in this study.

H_2_: Good ESG performance has a promoting effect on green technical efficiency but a restraining effect on green technological progress.

**Fig 1:**
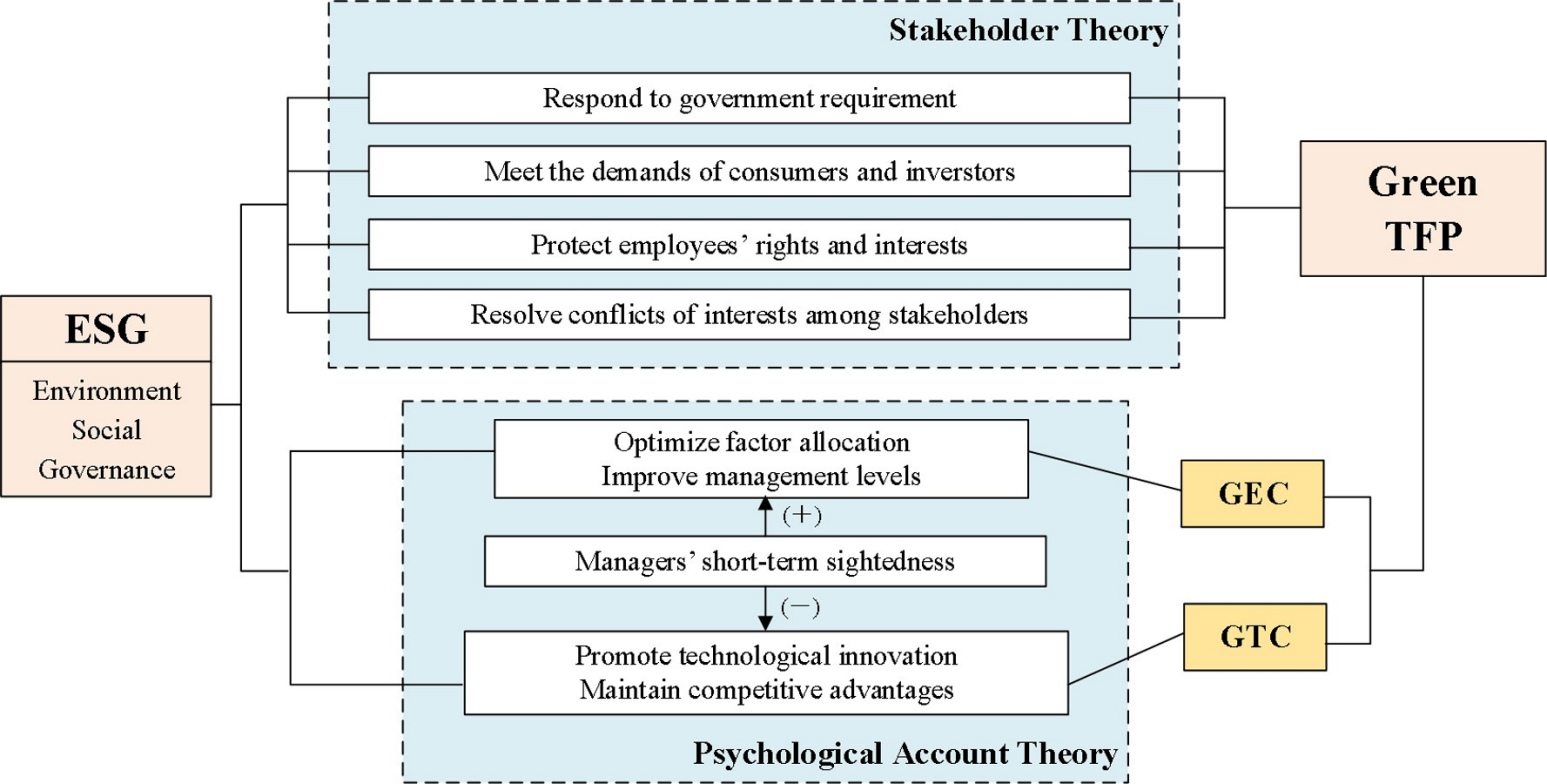
Theoretical framework for the impact of ESG on green TFP.

In summary, this study constructs a theoretical framework by combining stakeholder theory and psychological account theory (as shown in Fig 1). First, good ESG performance of a company can convey positive signals to stakeholders, enhance the confidence of stakeholder, establish good relationships with stakeholders, and obtain scarce resources from stakeholders, promoting the improvement of green TFP. In addition, the psychological account of managers leads to short-term loss aversion, and the promoting effect of ESG on green TFP mainly comes from the improvement of green technical efficiency (GEC), rather than green technological progress (GTC).

### 2.2 Samples and data

Based on data availability, this study used data for the period 2011 to 2021 that were sampled from selected Chinese A-share listed companies in the agriculture, forestry, animal husbandry, and fishery industries (category code A); mining industry (category code B); manufacturing industry (category code C); and electricity, heat, gas, and water production and supply industry (category code D). This selection was based on a number of reasons. (1) The “China Environmental Statistical Yearbook” only discloses the emissions data of the above four industries. (2) The statistical caliber of emission-related data in the “China Environmental Statistics Yearbook” has changed since 2011. To reflect the actual situation more accurately, we chose the longest possible time period to construct panel data and include more information. (3) Considering that the quarterly and semi-annual reports of listed companies in China do not require external auditing and cannot guarantee the authenticity of the data, we selected only the data disclosed in the annual reports for research. At the same time, we excluded research samples with special treatments and particular transfer as well as those with missing data, resulting in 14,421 valid samples. To control for the influence of extreme values on the model, all continuous variables were winsorized at the 1% and 99% levels.

### 2.3 Variables

#### 2.3.1 Explained variables

(1) Green TFP (GTFP)

TFP is a comprehensive reflection of the quality of economic development and management efficiency [36]. The concept is based on productivity, which refers to the ratio of output indicators to input indicators [37]. According to the quantity of input indicators, productivity can be divided into single factor productivity and total factor productivity. The former refers to the ratio of total economic output to a single factor input (such as labor input), while the latter (TFP) includes all input factors (including capital, labor, natural resources, etc.) and comprehensively reflects the efficiency of total input into output [38].

With the promotion of the green development concept, the impact of energy and environmental constraints on TFP cannot be ignored. However, the traditional TFP framework does not consider the negative externalities of economic growth, and it is difficult to meet the dual goals of economic growth and carbon emission reduction. Green TFP refers to the TFP of various input factors in the production process under the background of sustainable development. It not only includes traditional factors such as capital, labor, and natural resources, but also fully considers factors such as energy and environment [39], emphasizing a benign cycle between economic growth and environmental protection. In recent years, some scholars have begun to incorporate factors such as energy consumption and pollutant emissions into the TFP analysis framework to calculate green TFP [40,41], which better reflects the connotations of high-quality development.

There are two main methods for calculating TFP in existing studies: production function method and exponential method. The former is mainly based on the C-D production function, which can be traced back to Solow [42]. This method decomposes output into input growth and residual growth, and calculates the Solow residual through fixed effect model or stochastic effect model [43]. With the exogenous nature of technological progress, the questioning of Hicks’ neutrality assumption [44], and the subjective arbitrariness of parameter estimation [45], the limitations of production function method have been exposed. Caves et al. [46] constructed the Malmquist index based on the DEA theory, evaluating the efficiency of each of a set of comparable decision-making units [47], establishing a new theoretical framework to measure TFP.

The Malmqusit index method based on the DEA model has been widely used, but its application scenarios are still limited. It only considers the proportion of decrease (or increase) in input and output, and cannot distinguish the quality of output or the parts of relaxation improvement. Based on this, Tone [48] proposed the SBM model, which measures the inefficiency situation from both input and output perspectives, compensating for the shortcomings of traditional DEA models. In addition, Chung et al. [49] introduced the directional distance function into the computational framework and constructed the Malmquist–Luenberger productivity index (ML index) to address the problems of expected and unexpected outputs.

On the basis of existing research, this study used the SBM–ML model to calculate the level of green TFP. This study draws on the approach of Bruni et al. [50], and assumes that each company *k* is a decision-making unit (*k* = 1,2,⋯,*K*), and inputs *N* types of factors *x* = [*x*_1_,⋯,*x*_*K*_] ∈ *R*^*N*×*K*^, generates *M* types of expected output *y* = [*y*_1_,⋯,*y*_*K*_] ∈ *R*^*M*×*K*^, and *J* types of unexpected output *b* = [*b*_1_,⋯,*b*_*K*_] ∈ *R*^*J*×*K*^. The directional distance function that includes expected and unexpected outputs can be defined as:

$${\vec{D}}_{0}\left(x,y,b;g_{y},-g_{b}\right)=sup\left[\beta :\left(y+\beta g_{y},b-\beta g_{b}\right)\in p(x)\right]$$

where *g* is the direction vector, *g =* (*g*_*y*_, -*g*_*b*_), and *β* is the value of the distance function. The directional distance function of enterprise *k* in period *t* can be solved through the SBM model:

$${\vec{\text{D}}}_{0}^{\text{t}}\left(x_{i}^{t},y_{i}^{t},b_{i}^{t};y_{i}^{t},-b_{i}^{t}\right)=max\beta$$

$$s.t.\left\{\begin{array}{c} \sum_{\text{k}=1}^{K}z_{k}^{t}y_{km}^{t}\geq \left(1+\beta \right)y_{km}^{t},m=1,\cdots ,M\\ \sum_{k=1}^{K}z_{k}^{t}b_{kj}^{t}\geq \left(1+\beta \right)b_{kj}^{t},j=1,\cdots ,J\\ \sum_{k=1}^{K}z_{k}^{t}x_{kn}^{t}\geq x_{kn}^{t},n=1,\cdots ,N\\ z_{k}^{t}\geq 0,k=1,\cdots ,K \end{array}\right.$$

where $z_{k}^{t}$ is the weight. Based on the above steps, this study drew on the basic ideas of inclusive total factor productivity in macroeconomic research, and proposed that continuous inputs in the development process may generate both expected and unexpected outputs (such as pollution and unemployment). Therefore, we incorporated both expected and unexpected outputs into the green TFP indicator system. Using the slack-based measure (SBM) directional distance function [51,52] with a nondirectional and variable returns-to-scale model, we measured green TFP using the Malmquist–Luenberge (ML) productivity index (Eq 1):

$$GTFP_{t}^{t+1}={\left[\frac{1+{\vec{D}}_{0}^{t}\left(x^{t},y^{t},b^{t},y^{t},-b^{t}\right)}{1+{\vec{D}}_{0}^{t}\left(x^{t+1},y^{t+1},b^{t+1};y^{t+1},-b^{t+1}\right)}\times \frac{1+{\vec{D}}_{0}^{t+1}\left(x^{t},y^{t},b^{t};y^{t},-b^{t}\right)}{1+{\vec{D}}_{0}^{t+1}\left(x^{t+1},y^{t+1},b^{t+1};y^{t+1},-b^{t+1}\right)}\right]}^{\frac{1}{2}}$$ (1)

This study used the MaxDEA pro software to calculate the above process. If GTFP > 0, green TFP increases; if GTFP < 0, green TFP decreases. The input factor x includes three dimensions: labor input, capital input, and energy input, where y represents expected output and b represents unexpected output. The specific variable selection is shown in Table 1. Data sources mainly included the annual reports of listed companies, the database of the National Intellectual Property Administration, and the “China Environmental Statistics Yearbook.” To ensure the stability of the results, we conducted robustness tests by replacing the explanatory variable with a TFP indicator.

**Table 1:** Index system of green TFP.

Type	Code	Name	Calculation Method
Input	*x*	Labor Input	Number of employees
		Capital Input	Net value of fixed assets
		Energy Input	Converted quantity of standard coal [53] (Unit: ten thousand tons)
Output	*y*	Expected Output	Operating income of enterprises
	*b*	Unexpected Output	Comprehensively calculated based on the emission data of atmospheric pollutant SO_2_, chemical oxygen demand of water quality (COD), and solid waste discharge

(2) Green technical efficiency (GEC) and green technological progress (GTC)

At the micro level, the improvement of green TFP in enterprises is usually attributed to technological upgrading, improvement of production and factor allocation efficiency [54], economic scale effects, and economic growth driven by policy dividends and other factors [55], which is an important manifestation of enterprise heterogeneity. This study drew on the approach of Oh [56] by decomposing green TFP into two components: green technical efficiency (GEC) and green technological progress (GTC) [Eq 2]. GEC refers to the improvement of green TFP resulting from the continuous enhancement of resource allocation efficiency, such as the improvement of management efficiency and the optimization and reorganization of the production process (Eq 3), while the technological frontier remains unchanged. GTC refers to the advancement of the technological frontier, benefiting green TFP mainly through the new growth momentum brought about by the expansion of technological boundaries (Eq 4).


GTFP=GEC×GTC (2)

$$GEC_{t}^{t+1}=\frac{1+\vec{D_{0}^{t}}\left(x^{t},y^{t},b^{t};y^{t},-b^{t}\right)}{1+{\vec{D}}_{0}^{t+1}\left(x^{t+1},y^{t+1},b^{t+1};y^{t+1},-b^{t+1}\right)}$$ (3)

$$GTC_{t}^{t+1}={\left[\frac{1+{\vec{D}}_{0}^{t+1}\left(x^{t+1},y^{t+1},b^{t+1};y^{t+1},-b^{t+1}\right)}{1+\vec{D_{0}^{t}}\left(x^{t},y^{t},b^{t};y^{t},-b^{t}\right)}\times \frac{1+\vec{D_{0}^{t}}\left(x^{t+1},y^{t+1},b^{t+1};y^{t+1},-b^{t+1}\right)}{1+{\vec{D}}_{0}^{t+1}\left(x^{t},y^{t},b^{t};y^{t},-b^{t}\right)}\right]}^{\frac{1}{2}}$$ (4)

If GEC > 1, green technical efficiency of the enterprise has been improved; otherwise, it indicates a decline. Similarly, if GTC > 1, green TFP has been improved owing to green technological progress; while conversely, it indicates a decrease.

#### 2.3.2 Explanatory variable

ESG performance was the core explanatory variable. Following the research method of Song et al. [57], this study used the ESG ratings index released by Shanghai Huazheng Index Information Service Co., Ltd. (Huazheng) to measure the ESG performance of enterprises. Currently, stakeholders are no longer satisfied by focusing solely on financial reports [58]. The Huazheng ESG Index was sourced from the Wind database, which comprehensively covers annual social responsibility, sustainable development, and ESG reports publicly released by sample enterprises. It also collects ESG information from various channels, such as corporate websites, government regulatory agency websites, domestic and foreign media reports, and academic research reports. Negative factors and potential risks such as environmental penalties and negative business events of the sample enterprises were also considered [59]. The Huazheng ESG Index is widely recognized by the academic community. The higher the Huazheng ESG Index value, the better the ESG performance of the sample enterprise. To avoid bias arising from a single data source, we also used ESG indicators published by Bloomberg Consulting for the propensity score matching -difference-in-difference model (PSM–DID) analysis in the endogeneity test.

#### 2.3.3 Control variables

We followed the practices of Alon et al. [60] and Qi et al. [61] in introducing control variables in the corporate finance and corporate governance dimensions. The former consisted of leverage ratio (Lev), firm growth (Growth), capital intensity (CI), cash holdings (Cash), firm value (TobinQ), and individual stock return (IST). The latter consisted of proportion of independent directors (Inde), degree of equity balance (Z), and internal control defects (Incon). Due to the vulnerability of panel data analysis caused by excessive cross-sectional units and variables, we also controlled for industry and annual fixed effects. The definitions and calculation methods for these variables are listed in Table 2.

**Table 2:** Definitions and calculation methods of model parameters.

Type	Code	Calculation Method
Explained Variables	*GTFP*	ML productivity index based on directional distance function
	*GEC*	Productivity index decomposition—technical efficiency changes
	*GTC*	Productivity index decomposition—technological progress changes
Explanatory Variable	*ESG*	Huazheng ESG Index
Control Variables	*Lev*	Ratio of total liabilities to total assets
	*Growth*	Growth rate of operating income
	*CI*	Ratio of fixed assets to total assets
	*Cash*	Ratio of cash and cash equivalents to total assets
	*TobinQ*	Ratio of market value to total assets
	*IST*	Individual stock return considering the reinvestment of cash dividends
	*Inde*	Proportion of independent directors to total board members
	*Z*	Ratio of the shareholding ratios of the 2nd to 5th largest shareholders to that of the 1st largest shareholder
	*Incon*	Whether internal control deficiencies are disclosed in the report (disclosure: 1, otherwise: 0)
	*Industry*	Industry dummy variable
	*Year*	Year dummy variable

#### 2.3.4 Model construction

To validate the research hypotheses, examine the impact of ESG performance on the green TFP of enterprises, and decompose green technical efficiency and green technological progress, this study drew on the research of Xie et al. [62] and Cui et al. [63] to construct fixed effect regression models, as shown in Eqs (5)–(7). Among them, model (5) was used to test whether hypothesis 1 is valid, and models (6) and (7) were used to verify hypothesis 2. *GTFP*_*i*,*t*_ was measured using Eq 1, while *GEC*_*i*,*t*_ and *GTC*_*i*,*t*_ were measured using Eqs 3 and 4 respectively.

$$GTFP_{i,t}=\alpha_{0}+\alpha_{1}ESG_{i,t}+\sum \alpha_{2}X_{i,t}+\sum \alpha_{3}Y_{i,t}+\varepsilon_{i,t}$$ (5)

$$GEC_{i,t}=\beta_{0}+\beta_{1}ESG_{i,t}+\sum \beta_{2}X_{i,t}+\sum \beta_{3}Y_{i,t}+\theta_{i,t}$$ (6)

$$GTC_{i,t}=\gamma_{0}+\gamma_{1}ESG_{i,t}+\sum \gamma_{2}X_{i,t}+\sum \gamma_{3}Y_{i,t}+\mu_{i,t}$$ (7)

where *i* and *t* represent the individual and time dimensions of the research samples, respectively; *ε*, *θ*, and *μ* are the residuals of each regression model; and *X*_*i*,*t*_ and *Y*_*i*,*t*_ represent the control variables of the two dimensions of corporate finance and corporate governance, respectively. Based on the hypotheses, we predicted that the regression coefficients *α*_*1*_ and *β*_*1*_ would be significantly positive, and that *γ*_*1*_ would be significantly negative.

In addition, after confirming the above relationships, this study aimed to explore the underlying mechanisms and verify whether managers’ psychological accounts exist and how they function. Psychological account theory suggests that even if managers have ambitious goals and sustainable development visions, they will still divide their limited resources into different psychological accounts, resulting in tendentially short-sighted decisions when choosing green transformation strategies. As a result, good ESG performance only improves green technical efficiency within the existing technological frontier without truly expanding the boundaries of technological progress. This can be characterized as the green transformation type of “mending the situation before it is too late.” To verify the existence of this mechanism, we drew on Zhonglin and Baojuan [64] and constructed mediating effect models, as shown in Eqs (8)–(10).


$$GEC_{i,t}\left(GTC_{i,t}\right)=\delta_{0}+\delta_{1}ESG_{i,t}+\sum \delta_{2}X_{i,t}+\sum \delta_{3}Y_{i,t}+\epsilon_{i,t}$$ (8)

$$EnvM_{i,t}=\vartheta_{0}+\vartheta_{1}ESG_{i,t}+\sum \vartheta_{2}X_{i,t}+\sum \vartheta_{3}Y_{i,t}+\rho_{i,t}$$ (9)

$$GEC_{i,t}\left(GTC_{i,t}\right)=\varphi_{0}+\varphi_{1}ESG_{i,t}+\varphi_{2}EnvM_{i,t}+\sum \varphi_{3}X_{i,t}+\sum \varphi_{4}Y_{i,t}+\omega_{i,t}$$ (10)

where managers’ proactive environmental management indicator (EnvM) is the intermediary variable in the psychological account path. Following the approach of Yonggui and Xia [65], the measurement was conducted by examining whether there were relevant descriptions of environmental protection policies in companies’ environmental reports (such as the presence of environmental protection budgets and integration of environmental plans, strategies, or goals with marketing activities). If so, the value of EnvM_i,t_ was one; otherwise, it was zero.

## 3. Results and analysis

### 3.1 Baseline regression and mechanism test

Table 3 presents the impact of ESG performance on the green TFP of enterprises with (column 2) and without (column 1) the inclusion of control variables, as well as the decomposed components (GEC and GTC). The results in columns (1) and (2) demonstrate that regardless of the inclusion of control variables, ESG performance had a significant positive impact on companies’ green TFP (p < 0.01), supporting H_1_. Based on the data in columns (3) and (4), ESG performance had a significantly positive effect on GEC (p < 0.01) and a significantly negative effect on GTC (p < 0.01), supporting H_2_.

**Table 3:** Impact of ESG performance on green TFP and the decomposed components (GEC and GTC).

Variables	(1)	(2)	(3)	(4)
	GTFP (no controls)	GTFP	GEC	GTC
ESG	0.028^***^	0.029^***^	0.032^***^	−0.024^***^
	(3.265)	(3.386)	(3.582)	(−3.128)
Lev		0.035^***^	0.031^***^	0.014
		(3.980)	(3.470)	(1.630)
Growth		0.003	0.001	0.020^**^
		(0.360)	(0.094)	(2.355)
CI		−0.008	−0.003	−0.021^**^
		(−0.855)	(−0.341)	(−2.347)
Cash		0.001	0.006	−0.012
		(0.092)	(0.488)	(−1.261)
TobinQ		−0.014	−0.013	0.009
		(−1.491)	(−1.355)	(0.918)
IST		0.018	0.017	0.001
		(1.423)	(1.395)	(0.108)
Inde		0.015	0.014	0.016^**^
		(1.545)	(1.449)	(2.089)
Z		0.012	0.013	0.009
		(1.331)	(1.457)	(1.216)
Incon		0.034^*^	0.037^**^	0.001
		(1.830)	(2.005)	(0.044)
Constant	−0.057	−0.056	0.020	−0.587^***^
	(−0.633)	(−0.608)	(0.203)	(−6.669)
Industry/Year	Control	Control	Control	Control
*N*	14421	14421	14421	14421
*Adj*.*R*^*2*^	0.001	0.003	0.003	0.201

Notes: *t*-test values are in parentheses; ^***^, ^**^ and ^*^ represent significance at the 1%, 5%, and 10% levels, respectively. All variables were standardized to ensure dimensional uniformity.

To explore the mechanism behind these significant outcomes and examine the existence of managers’ psychological accounts, this study conducted a mechanism test using managers’ proactive environmental management indicator (EnvM) as the intermediary variable (Table 4). The short-term decision-making of managers caused by the psychological account had a significant explanatory effect of 34.4% (0.011/0.032) on the impact of ESG performance on GEC, and a significant explanatory effect of 20.8% (−0.005/−0.024) on the impact of ESG performance on GTC (Table 4, Panel A). The coefficients of all key variables of the mediating effect were significant at p < 0.1–0.01, confirming the existence of a psychological account path (Table 4, Panel B). In other words, good ESG performance can improve the green TFP of enterprises, but manager psychological accounts may turn long-term sustainable development into short-term decisions during implementation. Therefore, the improvement in ESG performance on green TFP was mainly achieved by improving green technical efficiency rather than by expanding technological boundaries.

**Table 4:** Psychological account path test results.

**Panel A: Reliability of statistical results on mediating effects**										
**Variables**	**Effects**			**Estimate**		**Standard error**		**Z**		**P**
GEC	Indirect effect			0.011		0.003		3.658		0.000
	Direct effect			0.021		0.009		2.297		0.022
	Total effect			0.032		0.009		3.734		0.000
GTC	Indirect effect			−0.005		0.003		−1.846		0.065
	Direct effect			−0.019		0.008		−2.391		0.017
	Total effect			−0.024		0.008		−3.190		0.001
**Panel B: Regression results of mediating effect**										
**Variables**		**(1)**	**(2)**		**(3)**		**(4)**		**(5)**	
		**GEC**	**GTC**		**EnvM**		**GEC**		**GTC**	
ESG		0.032^***^	−0.024^***^		0.630^***^		0.021^**^		−0.019^**^	
		(3.582)	(−3.128)		(40.337)		(2.192)		(−2.353)	
EnvM							0.018^***^		−0.008^*^	
							(3.210)		(−1.933)	
Lev		0.031^***^	0.014		0.288^***^		0.026^***^		0.016^*^	
		(3.470)	(1.630)		(18.826)		(2.882)		(1.882)	
Growth		0.001	0.020^**^		−0.046^***^		0.002		0.020^**^	
		(0.094)	(2.355)		(−3.435)		(0.189)		(2.313)	
CI		−0.003	−0.021^**^		0.316^***^		−0.009		−0.018^**^	
		(−0.341)	(−2.347)		(18.215)		(−0.902)		(−2.065)	
Cash		0.006	−0.012		0.029		0.005		−0.012	
		(0.488)	(−1.261)		(1.434)		(0.446)		(−1.235)	
TobinQ		−0.013	0.009		−0.095^***^		−0.012		0.009	
		(−1.355)	(0.918)		(−6.106)		(−1.187)		(0.846)	
IST		0.017	0.001		0.058^***^		0.016		0.002	
		(1.395)	(0.108)		(3.164)		(1.315)		(0.154)	
Inde		0.014	0.016^**^		−0.093^***^		0.016		0.015^**^	
		(1.449)	(2.089)		(−6.442)		(1.604)		(1.989)	
Z		0.013	0.009		−0.042^***^		0.014		0.009	
		(1.457)	(1.216)		(−2.854)		(1.542)		(1.172)	
Incon		0.037^**^	0.001		0.214^***^		0.033^*^		0.002	
		(2.005)	(0.044)		(6.723)		(1.808)		(0.149)	
Constant		0.020	−0.587^***^		0.602^***^		0.009		−0.582^***^	
		(0.203)	(−6.669)		(5.236)		(0.095)		(−6.610)	
Industry/Year		Control	Control		Control		Control		Control	
*N*		14421	14421		14421		14421		14421	
*Adj*.*R*^*2*^		0.003	0.201		0.217		0.004		0.201	

Notes: *t*-test values are in parentheses; ^***^, ^**^ and ^*^ represent significance at the 1%, 5%, and 10% levels, respectively. All variables were standardized to ensure dimensional uniformity.

### 3.2 Heterogeneity analysis

#### 3.2.1 Heterogeneity analysis of industry environmental risks

The demand for green, low-carbon, and circular development in China has gradually increased in the recent past. The environmental governance of local governments, environmental supervision of the public, and environmental attention of the capital market have greatly increased the governance costs of some industries with high environmental risks. This affects the green transformation strategy choices of enterprises in these industries and in turn the relationship between ESG performance and green TFP. Following Xin and Ying [66], we identified the environmental risks faced by the sampled enterprises. Enterprises in nine industries (nuclear power generation, hydroelectric power generation, water conservancy and inland port engineering construction, coal mining and washing, oil and natural gas mining, ferrous metal mining and dressing, non-ferrous metal mining and dressing, non-metallic mining and dressing, and other mining industries) were identified as high environmental risk enterprises. Companies in other sectors were classified as low environmental risk enterprises. Columns (1) to (3) of Table 5 present the results for enterprises with low environmental risks. The impacts of ESG performance on green TFP, GEC, and GTC were all highly significant (p < 0.01). However, effects were relatively weakly significant for enterprises with high environmental risk (columns 4–6). ESG performance still had a promoting effect on green TFP and GEC, while its impact on GTC was non-significantly positive. This indicates that when enterprises face high environmental risks, the role of ESG performance weakens, shifting from green transformation to environmental pollution control.

**Table 5:** Results of heterogeneity analysis of environmental risk.

Variables	Low environmental risk enterprises			High environmental risk enterprises		
	(1)	(2)	(3)	(4)	(5)	(6)
	GTFP	GEC	GTC	GTFP	GEC	GTC
ESG	0.026^***^	0.027^***^	−0.028^***^	0.068^**^	0.092^***^	0.006
	(2.899)	(2.941)	(−3.408)	(2.301)	(2.700)	(0.203)
Lev	0.039^***^	0.035^***^	0.019^**^	−0.018	−0.024	−0.073^**^
	(4.306)	(3.815)	(2.132)	(−0.444)	(−0.625)	(−2.279)
Growth	−0.000	−0.003	0.023^**^	0.023	0.025	0.005
	(−0.023)	(−0.288)	(2.455)	(0.940)	(1.212)	(0.278)
CI	−0.007	−0.001	−0.023^**^	0.012	0.012	0.063^**^
	(−0.672)	(−0.092)	(−2.461)	(0.410)	(0.360)	(2.216)
Cash	0.004	0.010	−0.011	−0.002	0.038	−0.048^*^
	(0.326)	(0.788)	(−1.084)	(−0.039)	(0.566)	(−1.758)
TobinQ	−0.011	−0.010	0.012	−0.065^**^	−0.058^**^	−0.054
	(−1.138)	(−1.008)	(1.136)	(−2.288)	(−2.117)	(−1.508)
IST	0.016	0.017	−0.002	0.009	−0.006	0.069^*^
	(1.254)	(1.274)	(−0.229)	(0.229)	(−0.167)	(1.676)
Inde	0.013	0.015	0.015^*^	0.037	−0.010	0.044
	(1.391)	(1.549)	(1.885)	(0.779)	(−0.246)	(1.379)
Z	0.017^*^	0.018^*^	0.006	−0.042^*^	−0.044^**^	0.039
	(1.790)	(1.939)	(0.807)	(−1.946)	(−1.964)	(1.505)
Incon	0.036^*^	0.040^**^	0.003	0.006	0.007	−0.064
	(1.856)	(2.065)	(0.158)	(0.095)	(0.097)	(−1.134)
Constant	−0.050	0.021	−0.603^***^	−0.013	0.070	−0.346^***^
	(−0.541)	(0.218)	(−6.825)	(−0.167)	(0.679)	(−3.069)
Industry/Year	Control	Control	Control	Control	Control	Control
*N*	13408	13408	13408	1013	1013	1013
*Adj*.*R*^*2*^	0.003	0.003	0.210	0.005	0.009	0.200

Notes: *t*-test values are in parentheses; ^***^, ^**^ and ^*^ represent significance at the 1%, 5%, and 10% levels, respectively. All variables were standardized to ensure dimensional uniformity.

#### 3.2.2 Heterogeneity analysis of enterprise life-cycle

Enterprises are organizations with lifecycle features similar to those of biological organisms, spanning a period from birth to death [67]. Enterprises at different life cycle stages exhibit significant differences in investment decision-making, R&D capabilities, and external environmental needs, which may affect managers’ psychological accounts and decision-making processes. This study adopted the Dickinson [68] cash flow model to classify the life cycles of enterprises into three categories: growth, maturity, and decline. The results in columns (1) and (2) of Table 6 show that ESG performance had the same effect on green TFP in the growth and maturity stages as in the original test. However, the relationship was no longer significant in the decline stage samples (column 3). We therefore further examined the impact of ESG performance on GEC and GTC in the decline-stage samples (columns 4 and 5).

**Table 6:** Life-cycle heterogeneity of enterprises in the growth, maturity, or decline stage.

Variables	Growth stage	Maturity stage	Decline stage		
	(1)	(2)	(3)	(4)	(5)
	GTFP	GTFP	GTFP	GEC	GTC
ESG	0.035^**^	0.031^**^	0.018	0.023	−0.032^*^
	(2.486)	(2.274)	(1.001)	(1.266)	(−1.886)
Lev	0.045^***^	0.024^*^	0.041^**^	0.024	0.032^*^
	(2.792)	(1.674)	(2.431)	(1.571)	(1.847)
Growth	−0.005	0.002	0.015	0.018	0.042^**^
	(−0.530)	(0.102)	(0.703)	(0.858)	(2.416)
CI	−0.003	−0.004	−0.023	−0.011	−0.061^***^
	(−0.198)	(−0.260)	(−0.999)	(−0.519)	(−2.950)
Cash	0.027	0.001	−0.018^*^	−0.015	−0.013
	(1.010)	(0.048)	(−1.698)	(−1.322)	(−0.829)
TobinQ	0.005	−0.031^**^	−0.017	−0.011	0.027
	(0.254)	(−2.091)	(−1.009)	(−0.682)	(1.442)
IST	0.018	0.011	0.020	0.029	−0.038^*^
	(0.792)	(0.631)	(0.865)	(1.226)	(−1.765)
Inde	0.005	0.032^*^	0.006	0.002	0.011
	(0.407)	(1.815)	(0.325)	(0.091)	(0.661)
Z	0.006	0.018	0.010	0.009	−0.022
	(0.467)	(1.148)	(0.549)	(0.481)	(−1.371)
Incon	−0.001	0.067^**^	0.031	0.032	0.043
	(−0.025)	(2.106)	(0.847)	(0.853)	(1.275)
Constant	0.050	−0.132	−0.033	0.042	−0.618^***^
	(0.285)	(−0.750)	(−0.311)	(0.396)	(−3.441)
Industry/Year	Control	Control	Control	Control	Control
*N*	6088	4998	3335	3335	3335
*Adj*.*R*^*2*^	0.001	0.006	0.003	0.004	0.239

Notes: *t*-test values are in parentheses; ^***^, ^**^ and ^*^ represent significance at the 1%, 5%, and 10% levels, respectively. All variables were standardized to ensure dimensional uniformity.

The impact of ESG performance on GEC was not significant, but a low-significance (p < 0.1) negative correlation with GTC was maintained. This was not due to the failure of the psychological account path, but rather precisely because the psychological account path had a stronger effect on decline-stage enterprises. In the decline stage, enterprises face reduced profit growth, deteriorating financial conditions, rigid systems, and hierarchical redundancies. Managers tend to make decisions with a negative attitude aimed at meeting the government’s minimum requirements for environmental protection, and the green transformation process shows the characteristics of “sewing and repairing.” Managers’ psychological accounts tend to allocate more resources to the maintenance of existing capabilities than to the improvement of green technical efficiency and technological progress. Therefore, decline-stage enterprises no longer pursue ways to improve their green TFP to cater to ESG ratings, and the connection between the two gradually decreases.

### 3.3 Endogeneity and robustness test

#### 3.3.1 Endogeneity test

We assumed that the mechanisms of data disclosure might have injected some biases into the sample selection and consequently caused endogeneity issues such as mutual causality between ESG performance and green TFP. To address this potential issue, we employed two endogeneity tests, the propensity matching score-difference-in-difference model (PSM–DID) and two-stage least squares (2SLS).

First, we used the ESG indicator (DESG) released by Bloomberg Consulting to conduct the PSM–DID test. The values for the experimental and control groups were determined as follows: if the ESG ratings for the enterprise were provided for the first time, the values for the enterprise in that year and in subsequent years were set to 1, otherwise to 0. Subsequently, all control variables were included in the logit model to determine the propensity score value, and paired samples that met the common support hypotheses were retained. The coefficient of the logit regression test was significant at p < 0.05, indicating a good fit. The ATT estimate was 0.047, and the t-test value was 2.39 (significantly greater than the critical value of 1.96). This indicates that a company’s ESG performance significantly affected green TFP. After matching, 9,128 samples were obtained. We used matched data for the DID test, the results of which are presented in Table 7. The impacts of DESG on green TFP, GEC, and GTC were consistent with the original results, which supports the proposed hypotheses and indicates the absence of sample selection biases that may lead to endogeneity issues.

**Table 7:** PSM–DID endogeneity test results.

Variables	(1)	(2)	(3)
	GTFP	GEC	GTC
DESG	7.069^***^	7.513^***^	−0.055^***^
	(3.577)	(3.936)	(−4.168)
Lev	7.902	6.021	0.082^**^
	(1.471)	(1.137)	(2.028)
Growth	−0.957	−1.481	0.030^**^
	(−0.540)	(−0.897)	(2.244)
CI	−8.719	−6.758	−0.026
	(−1.202)	(−0.932)	(−0.498)
Cash	−0.704	−0.637	0.128^*^
	(−0.078)	(−0.072)	(1.669)
TobinQ	−1.818^**^	−1.782^**^	0.006
	(−2.159)	(−1.998)	(0.855)
IST	1.865	0.990	0.003
	(0.637)	(0.366)	(0.188)
Inde	29.680	30.003	0.175
	(1.447)	(1.439)	(1.334)
Z	5.041^**^	4.535^**^	0.012
	(2.569)	(2.427)	(0.964)
Incon	1.317	1.659	−0.004
	(0.624)	(0.809)	(−0.274)
Constant	0.578	7.429	0.703^***^
	(0.042)	(0.538)	(7.242)
Industry/Year	Control	Control	Control
*N*	9128	9128	9128
*Adj*.*R*^*2*^	0.004	0.004	0.202

Notes: *t*-test values are in parentheses; ^***^, ^**^ and ^*^ represent significance at the 1%, 5%, and 10% levels, respectively. All variables were standardized to ensure dimensional uniformity.

Subsequently, instrumental variables were selected to conduct the 2SLS test to examine possible endogeneity issues caused by mutual causation. Given the potential strong association between the environmental (E) sub-score in the Huazheng ESG Index and green TFP, the social (S) and governance (G) sub-scores have a strong connection with the explanatory variable, which is unrelated to the disturbance term of the dependent variable. We selected the S and G sub-scores as instrumental variables for testing. The Kleibergen–Paap rk LM test was significant (p < 0.01), rejecting the hypothesis of underidentification, and the Hansen J test was p > 0.1, indicating that all instrumental variables in the model were exogenous. The final results are shown in Table 8, where column (1) represents the first-stage regression with good explanatory power for the instrumental variables on the explanatory variable. The results of the second-stage regressions in columns (2)–(4) are consistent with the main tests, demonstrating the robustness of the findings after controlling for endogeneity.

**Table 8:** Results of the 2SLS endogeneity test.

Variables	(1)	(2)	(3)	(4)
	ESG	GTFP	GEC	GTC
S	0.367^***^			
	(168.288)			
G	0.469^***^			
	(170.990)			
ESG		0.520^***^	0.527^***^	−0.002^**^
		(3.528)	(3.512)	(−2.104)
Lev	1.239^***^	17.055^***^	14.434^***^	0.055^*^
	(15.014)	(4.012)	(3.481)	(1.734)
Growth	−0.114^***^	0.551	0.134	0.027^**^
	(−4.264)	(0.367)	(0.094)	(2.389)
CI	0.944^***^	−5.046	−1.995	−0.099^**^
	(8.782)	(−0.860)	(−0.342)	(−2.371)
Cash	−0.046	0.218	1.270	−0.021
	(−1.012)	(0.084)	(0.488)	(−1.303)
TobinQ	−0.161^***^	−1.011	−0.922	0.006
	(−13.294)	(−1.468)	(−1.358)	(1.009)
IST	0.193^***^	3.279	3.204	0.001
	(5.396)	(1.415)	(1.398)	(0.051)
Inde	−1.198^***^	25.119	23.751	0.209^**^
	(−4.605)	(1.532)	(1.447)	(2.025)
Z	−0.067^***^	1.911	2.034	0.012
	(−2.681)	(1.338)	(1.458)	(1.247)
Incon	−0.009	3.099^*^	3.351^**^	0.000
	(−0.309)	(1.828)	(2.006)	(0.029)
Constant	8.719^***^	−36.292^**^	−30.323^**^	0.916^***^
	(25.129)	(−2.380)	(−1.985)	(8.679)
Industry/Year	Control	Control	Control	Control
*N*	14421	14421	14421	14421
*Adj*.*R*^*2*^	0.909	0.003	0.003	0.201

Notes: *t*-test values are in parentheses; ^***^, ^**^ and ^*^ represent significance at the 1%, 5%, and 10% levels, respectively. All variables were standardized to ensure dimensional uniformity.

#### 3.3.2 Robustness test

Since ESG performance may have a wider impact on total factor productivity (TFP), this study replaced the green TFP indicator of the main test with the TFP indicator for robustness tests, and used the GMM, LP, and OP-methods for calculation and regression analysis. Columns (1)–(3) of Table 9 show that the results remained consistent with the original test after replacing the explained variable. In addition, compared with heavily polluting enterprises, the improvement effect of ESG performance on green TFP may be weaker in non-heavily polluting enterprises. Following Qingyuan and Zehua [69], we selected non-heavily polluting enterprises for robustness testing based on the list of heavily polluting enterprises formulated in the “Guidelines for Environmental Information Disclosure of Listed Companies” released by China’s Ministry of Environmental Protection in 2010. The results for these enterprises are presented in columns (4)–(6). The direction and significance level of the impact of ESG performance on green TFP, GEC, and GTC remained consistent with the main test, confirming the robustness of the conclusions.

**Table 9:** Robustness test results.

Variables	Replacement of explained variable			Non-heavily polluting enterprises		
	(1)	(2)	(3)	(4)	(5)	(6)
	TFP_GMM	TFP_LP	TFP_OP	GTFP	GEC	GTC
ESG	0.076^***^	0.202^***^	0.225^***^	0.031^***^	0.036^***^	−0.040^***^
	(13.562)	(32.620)	(36.364)	(2.660)	(3.117)	(−3.720)
Lev	0.222^***^	0.390^***^	0.420^***^	0.035^***^	0.028^**^	−0.008
	(33.082)	(53.624)	(57.883)	(2.919)	(2.341)	(−0.662)
Growth	−0.005	−0.033^***^	−0.051^***^	0.001	−0.002	0.016
	(−0.643)	(−5.236)	(−8.415)	(0.060)	(−0.242)	(1.548)
CI	−0.484^***^	−0.063^***^	−0.132^***^	−0.017	−0.018	−0.015
	(−69.185)	(−8.541)	(−18.029)	(−1.291)	(−1.341)	(−1.196)
Cash	0.030^***^	0.041^***^	0.056^***^	−0.004	−0.004	−0.009
	(4.862)	(6.085)	(8.368)	(−0.276)	(−0.288)	(−0.751)
TobinQ	−0.079^***^	−0.254^***^	−0.267^***^	−0.012	−0.008	−0.003
	(−11.496)	(−30.670)	(−32.049)	(−0.926)	(−0.590)	(−0.202)
IST	0.056^***^	0.130^***^	0.140^***^	0.017	0.015	−0.012
	(7.821)	(16.464)	(17.557)	(0.987)	(0.914)	(−0.925)
Inde	0.005	0.009	0.006	0.016	0.012	0.023^**^
	(0.957)	(1.581)	(0.933)	(1.401)	(1.002)	(2.321)
Z	−0.026^***^	−0.030^***^	−0.027^***^	0.002	0.000	0.011
	(−4.967)	(−5.188)	(−4.812)	(0.231)	(0.016)	(1.074)
Incon	0.014	0.073^***^	0.077^***^	−0.001	0.007	0.012
	(1.243)	(5.913)	(6.301)	(−0.047)	(0.291)	(0.572)
Constant	−0.383^***^	−0.493^***^	−0.397^***^	−0.050	0.022	−0.625^***^
	(−9.864)	(−10.764)	(−8.241)	(−0.524)	(0.218)	(−6.661)
Industry/Year	Control	Control	Control	Control	Control	Control
*N*	19341	19341	19341	9001	9001	9001
*Adj*.*R*^*2*^	0.491	0.405	0.392	0.001	0.002	0.234

Notes: *t*-test values are in parentheses; ^***^, ^**^ and ^*^ represent significance at the 1%, 5%, and 10% levels, respectively. All variables were standardized to ensure dimensional uniformity.

## 4. Discussion and conclusions

Against the background of the international consensus on green, low-carbon, and sustainable development, ESG reports have gradually gained increasing attention and are now regarded as the secondary financial reports of enterprises. China’s ESG system is still in the exploratory stages and does not require companies to disclose ESG reports. Third-party institutions such as securities firms conduct comprehensive assessments of various aspects of corporate performance. Whether ESG performance affects the green transformation strategies of enterprises is a key issue in current academic research. Therefore, this study combined stakeholder theory and psychological account theory to conduct in-depth research on the relationship between ESG performance and green TFP and the underlying transmission mechanism, using Chinese A-share listed companies from 2011 to 2021 as research samples.

The results demonstrate that good ESG performance can significantly enhance a company’s green TFP and that this effect is more pronounced in industries with lower environmental risks and in enterprises in the growth and maturity stages. Furthermore, the results of the mechanism test confirm the existence of a psychological account path, as the improvement of green TFP by ESG performance mainly derived from the improvement of green technical efficiency rather than the expansion of green technological boundaries. This is because the presence of psychological accounts leads managers to exhibit psychological aversion to short-term losses, and to base their long-term investment decisions on the evaluation of short-term returns. Consequently, they may be more inclined to improve short-term and more efficient green technical efficiency to improve green TFP, while avoiding green technological progress that can drive the expansion of the technological frontier. This finding provides a clear explanation for the contradictory conclusions of existing research on the relationship between ESG performance and green transformation.

The conclusions of this study have several practical implications. First, enterprises should fully recognize the enhancing effect of ESG performance on green TFP, establish green development concepts and social responsibility awareness, improve ESG ratings, and fully demonstrate their comprehensive capabilities for sustainable development, thereby absorbing more strategic resources from the market and stakeholders and creating conditions for the improvement of green TFP. As noted, managers’ psychological accounts may have a short-sighted effect when facing the soft regulation of ESG ratings; as strategic decision-makers in enterprises, managers should recognize that, compared with the improvement of green technical efficiency, green technological progress will be more meaningful for the development of both the enterprise itself and the entire economy and society. Managers should lead enterprises to choose high-quality green transformation strategies, form sustainable competitive advantages, and transform the growth of green TFP from a reliance on technical efficiency improvement alone to a dual reliance on technological boundary expansion and technical efficiency improvement.

Second, the relevant government agencies should accelerate the construction of an ESG system that is in line with international standards and suitable for China’s national conditions; improve the institutional construction of ESG in information disclosure, evaluation, and management; and formulate differentiated policies based on the environmental risks faced by enterprises and their life-cycle characteristics. They should guide and regulate enterprises to actively fulfill ESG responsibilities and promote the implementation of sustainable development strategies at the micro level. Simultaneously, the ESG rating system and information release mechanism should be improved, and the synergy between government regulations and professional supervision should be strengthened. The effectiveness of ESG ratings cannot be achieved without accounting for the driving force behind enterprises’ green development demands. To fundamentally improve the green TFP of enterprises, the government should encourage social rating agencies to continuously improve their ability to explore the long-term value of enterprises; fully play the positive role of soft market regulation, such as securities analysts, auditors, and the media; effectively constrain and correct the psychological accounts of managers; form a positive feedback loop mechanism for ESG performance; and cultivate managers’ green development concepts.

## Supporting information

S1 Data (XLS)
